# Assessment of enteroviruses from sewage water and clinical samples during eradication phase of polio in North India

**DOI:** 10.1186/s12985-018-1075-7

**Published:** 2018-10-16

**Authors:** Sarika Tiwari, Tapan N. Dhole

**Affiliations:** 1 Department of Microbiology (Virology Section), Sanjay Gandhi Post Graduate Institute of Medical Sciences (SGPGIMS), Lucknow, Uttar Pradesh 226014 India; 2 Department of Microbiology (Virology lab), Rajendra Institute of Medical Sciences (RIMS), Ranchi, JH India

**Keywords:** Acute flaccid paralysis, Environmental surveillance, Phylogenetic analysis, Sewage water

## Abstract

**Background:**

The *Enterovirus* (EV) surveillance system is inadequate in densely populated cities in India. EV can be shed in feces for several weeks; these viruses are not easily inactivated and may persist in sewage for long periods. Surveillance and epidemiological study of EV-related disease is necessary.

**Methods:**

In this study, we compare the EV found in sewage with clinically isolated samples. Tissue culture was used for isolation of the virus and serotype confirmed by enterovirus neutralization tests.

**Results:**

We found positive cases for enterovirus from clinical and sewage samples and identified additional isolates as echovirus 9, 11, 25 & 30 by sequencing.

**Conclusion:**

There is a close relation among the serotypes of enterovirus shed in stools and isolated from the environment but few serotypes which were detected in sewage samples were not found clinically and the few which were detected clinically not found in sewage because some viruses are difficult to detect by the cell culture method.This study will be helpful for the researchers who are working on polio and nonpolio enterovirus especially in the countries which are struggling for polio eradication.

## Background

*Enterovirus* can be transported in the environment through groundwater, estuarine water, seawater, rivers, aerosols emitted from sewage treatment plants, insufficiently treated water, drinking water, and private wells that receive treated or untreated waste water either directly or indirectly. These viruses are usually transmitted via the fecal-oral route and primarily infect and replicate in the gastrointestinal tract of the host [1]. In addition to causing acute diseases, EV are of public health concern because of the low infectious dose needed to cause disease [2].

*Enterovirus* is nonenveloped, single-stranded, positive-sense viruses belonging to the family *Picornaviridae*. They include poliovirus, coxsackievirus (CB) groups A and B, echoviruses (E), and the *enterovirus*. Till date, more than 100 serotypes have been identified [3] including more than 70 *enterovirus* serotypes have been identified in humans [4]. EV may cause various symptoms, varying from asymptomatic infection to gastroenteritis, myocarditis and aseptic meningitis [5]. Human echovirus and coxsackievirus display a changing pattern of dominant serotypes in both sewage and clinical isolates, Echovirus 6, 19, 3, and coxsackievirus B4, B5, A9 have successively became the most common serotypes [6]. In the environment, enterovirus can survive under a wide pH range (pH 3 to 10) and for extended periods at low temperature [7]. Some of the EV have been isolated from sewage water, which were *coxsackievirus* types B-3, B-4, and B-5 and E1, E7, and E11 [8]. Interestingly, these isolations are related to reports of isolation of the same strains during the same period in clinical samples. In general, enterovirus show great potential to be used as water quality indicators to assess the risks associated with infectious virus transmission as well as to identify the dominant source of fecal contamination in water [8]. Assessment of enterovirus plays an important role in exploring the circulation of these viruses in the community. Earlier studies from 1974 to 1977 in U.K., 1979–1981 in Canada, from 1994 to 2002 in Milwaukee, Wisconsin, USA, and 2000–2007 in Finland [6, 8–10] comparing sewage and clinical EVs reported and demonstrated a similarity of clinical and sewage EV. Environmental surveillance should be considered a complementary assessment tool to trace prevalent and negligible enterovirus circulating in the human population.

## Methods

### Sewage sample collection

Sewage sample (approx one liter) was collected and stored in sterile glass bottle from the collection site by directly dipping into the sewage effluent. The outer surface of the bottle was rinsed with 2% sodium hypo chloride solution, placed in a cool box, and transported to the laboratory within 1 h after collection from sewage treatment plant, Daulatganz Lucknow, U.P., India (which was almost 25 km. away from the institute, covers entire north, west and central zone of Lucknow city). It covers a population of approximately 10, 00,000 and is the main sewage treatment plant of the area. The bottle was frozen at − 20° c, until the process of virus isolation.

### Clinical sample collection

Stool samples were collected from suspected AFP (Acute Flaccid Paralysis) patients from Sanjay Gandhi Post Graduate Institute of Medical Sciences (clinical samples come from north, west and central zone of Lucknow city), Lucknow, Uttar Pradesh, India.

### Sewage sample processing

Sewage samples were processed for virus isolation as soon as they were received in the laboratory. The sewage sample was concentrated by two-phase concentration method on the same day as described previously [11]. In brief, the pH of the sample was adjusted to 7.2 and the sample was centrifuged at 5000 g for 30 min at 4 °C to pellet the solids. 500 ml of clarified sewage was mixed with defined amounts of two polymers, dextran and 15% polyethylene glycol (15% PEG6000). The homogenous mixture obtained by vigorous shaking is left to stand overnight at 4 °C in a separating funnel. This allows the polymers to separate in two distinct layers (phases) in the funnel. Enterovirus accumulates in the smaller bottom layer and/or at the boundary between the layers (interphase). The bottom layer and interphase were collected drop-wise. The pellet from the initial centrifugation was suspended in this concentration, and treated with chloroform, then centrifuged at 5000 g for 30 min at 4 °C and assayed for the presence of virus. The concentrated virus suspension was stored at − 20 °C until used for virus isolation [12]. This method was published in a study which compared three different methods, including direct isolation, centrifugation and two phase separation, and results of their study suggest that the two phase separation method is the best for maximum virus yield [13].

### Stool sample processing

Stool sample processing was performed according to WHO protocol. In brief, Stool suspensions were prepared by adding 5 ml of phosphate-buffered saline, 1 g of glass beads (Corning Inc., Corning, NY), antibiotics (Penicillin and streptomycin, amphotericin B from GIBCO, USA). 1 ml chloroform (LAB CHEM) to 1 g of stool samples, shaked the mixture vigorously for 20 min in a mechanical shaker, and centrifuged at 1500 x g for 30 min at 4 °C [14]. Supernatant was kept at − 20 °C until the inoculation on cell monolayer.

### Cell culture

Human rhabdomyosarcoma (RD) and L20B (transgenic) cells were obtained from the Center for Disease Control and Prevention, Atlanta, GA, USA. Minimum essential media (MEM) of Earle’s salt solution and fetal bovine serum (FBS) were purchased from (Sigma Aldrich, USA). All cell culture media contained HEPES buffer, L-glutamine, sodium bicarbonate penicillin, streptomycin, amphotericin B from GIBCO, USA. Cell cultures were grown at 37 °C in incubators with supply of 5% CO_2_. Cell cultures for virus isolation were grown in 25cm^2^ plastic flasks (Costar, Corning, N.Y.) with 10% FBS (MEM) and maintained at 2% FBS (MEM) containing 7 ml MEM, and tissue culture tube with 1.5 ml (2% FBS).

### Inoculation of sewage samples

The extracted concentrate was inoculated on fresh monolayer cultures of L20B and RD cells in 50 ml (25 cm^2^) flasks. The cultured flasks were incubated at 37 °C and examined at every 24 h for cytopathic effect (CPE). Samples which showed CPE were frozen and thawed two times, then re-passaged on new cells. Samples were observed for CPE up to 7 days before being considered negative. Samples showed CPE were stored for confirmation through serotyping.

### Inoculation of stool samples

The supernatant (0.2 ml each) of stool suspension were inoculated in both RD and L20B cell lines (90% monolayer with maintenance medium.). The cells after inoculation were incubated at 36 °C and observed under an inverted microscope every day up to 1 week. Furthermore, re-passaged cultures were analyzed for 1 week. Negative cultures were examined at least for 14 days before being discarded. The cultures positive for virus growth (showed + 3 CPE, i.e. 75% lysed cells) were stored at − 20 °C for further analysis.

### Identification of enteroviruses

Samples showed CPE in both L20B and RD were considered as positive for the polio virus (PV) [15, 16]. However, some of the non-polio enteroviruses (NPEV) were those that showed CPE only in RD cells.

### Micro-neutralization assay

The kit was supplied by RIVM, Bilthoven, Netherlands, which included eight antiserum pools (A to G) and Cox B pool. Initially cell culture infective doses (CCID_50_) of the virus isolate were determined by serial dilution and 100 CCID_50_ was used for neutralization of the isolates with cross absorbed antiserum pools. Isolates were diluted to 10^− 3^ and 10^− 4^ respectively, and 0.05 ml of virus dilution was added to 0.05 ml of antiserum. Virus isolates were allowed to neutralize with antiserum for at least 1 h at 37 °C in 96 well micro titer plates (Coster, Corning USA). Then 0.1 ml of L20B cell suspension (5 × 10^5^ cells ml^− 1^) was added to each well. Plates were observed for cytopathic effect and neutralization pattern after 24 h for 3–5 days. The EV serotype was identified by the neutralization pattern (two neutralized wells for each serotype) according to manufacturer’s instruction [17].

### Virus isolation

Each positive viral suspension of sewage and clinical (which showed CPE in cell culture) was used to inoculate in four 25-cm2 culture flasks of freshly confluent monolayers of L20B cells*.* The volume of inoculum were used as 0.1, 0.2, 0.3, and 0.5 ml one flask each. The cell control flask was inoculated with 0.3 ml of PBS. After virus adsorption by incubation at 45 min, the inoculum was removed, the cells were washed with 5 ml of PBS, and 7 ml of culture medium (minimal essential medium containing 2% fetal bovine serum) was added to each flask. The flasks were incubated at 36 °C for 7 days and examined daily under a microscope for virus-induced cytopathic effects. Simultaneously, 0.1 ml of 1:10 dilution of virus concentration was used to inoculate RD cell monolayer and cells were grown in tissue culture tubes. The tubes were incubated at 36 °C for up to 7 days and examined under an inverted microscope to observe cytopathic effects.

### RT-PCR for enterovirus

Isolates grown in cell culture were freeze-thawed three times, centrifuged at 16000 g for 10 min, 5 μl of supernatant was taken in a fresh tube and 10 μl of distilled water was added for dilution. One-step RT–PCR is used to amplify the product [18]; For each reaction, 50 pmol each of primer E-1 and primer E-2 (pan-enterovirus primer provided by CDC, Atlanta, GA, targeting the conserved 5’ UTR: E-1(sense)–5′-ACACGGACACCCAAAGTAGTCGGTTCC 3′ E-2(Antisense)– 5′-TCCGGCCCCTGAATGCGGCTAATCC 3′) were used [19]. Buffer containing 100 mM Tris-HCl, 15 mM MgCl_2_, 500 mMKCl, pH 8.3 and 1 μl of diluted samples was added was incubated at 95 °C in thermocycler (ABI 9700 PCR Thermal cycler Geneamp) for 5 min and immediately chilled on ice. Five μl of enzyme buffer containing 0.7 μl of 1 M DTT (dithiothreitol), 6.9 μl of 40 Uμl^− 1^ Protector RNase inhibitor (Roche Applied Science), 4.5 μl of 20Uμl^− 1^ Reverse Transcriptase avian myeloblastosis virus (Roche Applied Science), 13.7 μl of 5Uμl^−1^Taq polymerase (Roche Applied Scsience) and 1.0 μl of 10 mM dNTPs (Roche Applied Science). Non-infectious RNA used as positive control and culture supernatant from uninfected cells was used as negative reagent control.

### RT-PCR product purification

Electrophoresis of RT-PCR product was performed in 1.5% low melting agarose gel. DNA was eluted from gel piece using QIAGEN gel extraction columns (QIAGEN, Chatsworth, CA, USA). DNA was eluted in 30 μl of Tris EDTA (TE) buffer (pH 8.0.)

### PCR product sequencing

PCR products were analyzed for the selected E9, 11, 25, and 30, isolates purified from 2.5% Nusieve agarose gel extraction kit according to manufacturer’s instructions (QIAGEN, Chatsworth, CA, USA). BLAST pairwise alignments were used for data analysis.

### Alignment and phylogenetic analysis

Nucleotide BLASTn analysis (http://www.ncbi.nlm.nih.gov/BLAST) was used to identify related genes of the viruses, and the reference sequences were obtained from the GenBank. The ClustalX version 2.0.12 was used to perform multiple nucleotide alignment and tree was constructed (Njplot Version 2.3) [20], the neighbor-joining method according to the distances between all pairs of sequences in a multiple alignment. The confidence of sequence clustering was evaluated by bootstrapping (1000 replicates) [21] in Fig.2.

## Results

**Fig. 1:**
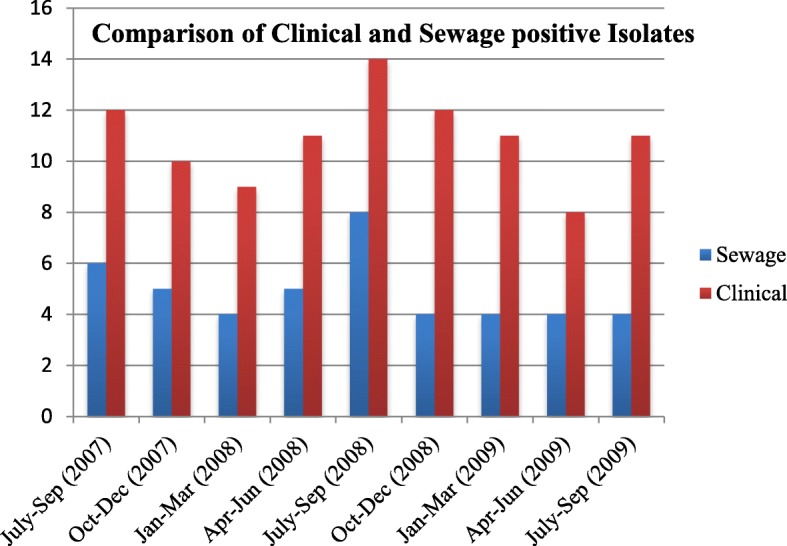
Comparison of number of positive enterovirus cases- isolated clinically and from sewage during the period of study

There were 400 and one samples of patients with suspected acute flaccid paralysis and 109 sewage samples were collected during July 2007–September 2009. 98 out of 401 and 44 out of 109 were detected positive for enterovirus in clinical and sewage samples respectively (Table 1) by cell culture and followed by serotyping and RT- PCR. The peak season for maximum detection of EV was from July to September in each year, although the occurrence of echovirus was observed throughout the year (Fig.1).

**Table 1:** Number of typed and untyped enteroviruses isolated from Sewage and Patients

Year	No. of EVs isolated from sewage				Total	No. of EVs isolated from patients				Total
	PV	CBV	Echo virus	UT		PV	CBV	Echo virus	UT	
2007	01	02	06	02	11	04	03	11	04	22
2008	04	01	11	05	21	09	08	18	11	46
2009	02	02	04	04	12	08	05	04	13	30
Total	07	05	21	11	44	21	16	33	28	98

*PV* Poliovirus, *CBV* Coxsackie B virus, *UT* Untyped virus

### Clinical isolates analysis

Out of 98 samples of EV, echoviruses were 33.6% [22], CB – 16.3% [19], PV - 21.4% [23] and Untypeable – 28.5% [24]. The maximum numbers of isolate were detected during the month of July–September 2008, in which the typed NPEV were 54.6% and untyped NPEV were 45.4% (Table 2).

**Table 2:** Number of typed and untyped non polio enteroviruses isolated from Sewage and Patients

Year	No. of NPEV isolated from sewage		Total	No. of NPEV isolated from Patient		Total
	No. of typed NPEV	No. of untyped NPEV		No. of typed NPEV	No. of untyped NPEV	
2007	08	02	10	14	04	18
2008	12	05	17	26	11	37
2009	06	04	10	09	13	22
Total	26	11	37	49	28	77

*PV* Poliovirus, *CBV* Coxsackie B virus, *UT* Untyped virus

### Sewage isolates analysis

**Fig. 2:**
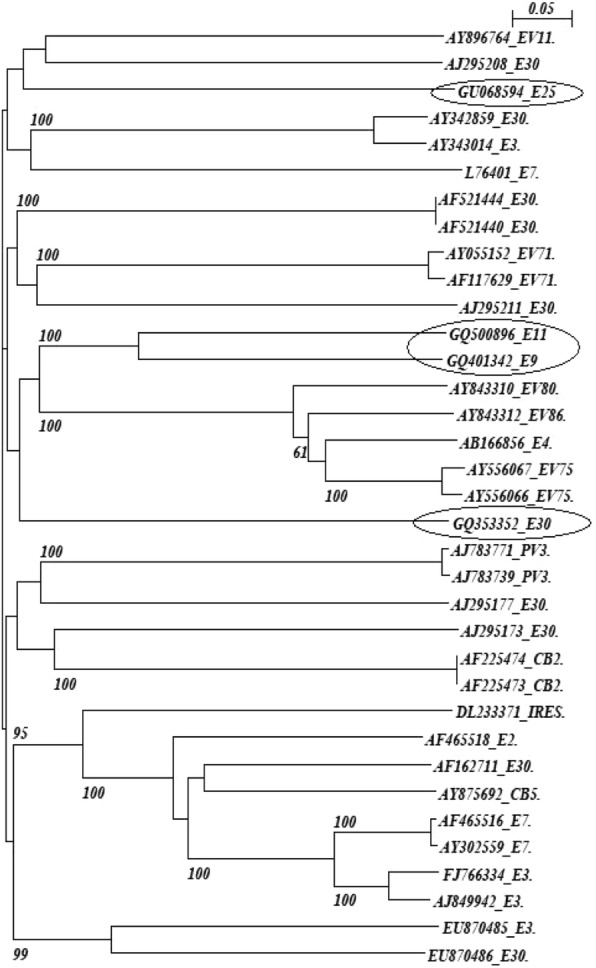
Phylogenetic tree of EV isolates from this study (indicated by circles) and other reference strains. Scale bar indicates nucleotide substitutions per site

Human echoviruses- 47.72% [23], coxsackieviruses B- 11.4% [5], PV- 15.9% [7] and Untypeable- 25.0% [12] were found out of 44 EV positive isolates. During July to September, 2008 NPEV and UT were predominant, which accounted for 83.3 and 16.6% respectively, whereas from October 2008 to July 2009 the number of EV isolates remained constant. Seven polioviruses were isolated from sewage, with no positive poliovirus being detected in the period of July to September 2007, January to March 2008, January to March 2009 and April to June 2009 respectively. Untyped samples were analyzed by RT-PCR using pan-EV primers (CDC, Atlanta, GA), and followed by sequence analysis. The GenBank accession numbers for these isolates are GQ401342, GQ500896, GU068594 and GQ353352 respectively. NPEV serotypes showed 95 to 100% homology with other enteroviruses (Fig. 2).

### Comparison between clinical and sewage EVs

Comparison of EV isolated quarterly from sewage and clinical cases were shown in Table 3. During July to September 2007, UT accounted for 25.5% of the clinical and 33.3% of the sewage isolates, while during October to December 2007 CB accounted for 30.0% of the clinical cases and 20.0% of the sewage isolates. During the month of April to June 2008, UT isolates accounted for 36.3% of the clinical cases and 20.0% of the sewage isolates; however from July to September 2008 UT isolates accounted for 35.7% of clinical infection and 12.5% of the sewage isolates. During the month from April to June 2009, the UT isolates accounted for 50.0% in clinically and 75.0% of the sewage isolates. However, from July to September 2009 UT isolates accounted for 36.4% of clinical infection and 50.0% of the sewage isolates. But the most common clinical echovirus for a particular year is not always the most common echovirus in sewage, from July to September 2007, January to March 2008, and October to December 2008. From July to September 2007, E4 was most frequently detected clinically (16.6% cases) but were not found in sewage, and during the month January to March 2008, UT isolates were more common in clinical samples (11.1% of the cases) as well as in sewage samples (40.0% of the isolates) whereas, E9, E12, E25, E30 and CB were more frequently isolated clinically but were not found in sewage samples. Echovirus 13 and E30 were present in sewage samples, but were not appearing in clinical samples during the month of October to December 2008.

**Table 3:** Enteroviruses Comparison Sheet: Positive Clinical isolates/ Positive Sewage Isolates

Percentage of Clinical virus Isolates / Percentage of sewage Isolates									
Virus	2007		2008				2009		
	July- Sep.	Oct- Dec.	Jan.- March	April–June	July- Sep.	Oct. – Dec.	Jan.- March	April–June	July- Sep.
N/N	12/6	10/5	9/4	11/5	14/8	12/4	11/4	8/4	11/4
E3	0/0	0/0	0/25.0	0/0	0/0	0/0	0/0	0/0	0/0
E4	16.6/0	0/0	0/0	9.0/0	0/0	0/0	0/25.0	0/0	0/0
E6	16.6/16.6	0/0	0/0	0/0	0/0	0/0	0/0	0/25.0	0/0
E7	0/0	10.0/0	0/25.0	0/0	7.1/12.5	0/0	9.0/0	0/0	0/0
E9	8.3/33.3	10.0/0	11.1/0	9.0/20.0	0/0	8.3/0	0/25.0	0/0	0/0
E11	8.3/0	20.0/20.0	0/0	0/0	14.3/12.5	8.3/0	0/25.0	0/0	0/0
E12	8.3/0	0/0	11.1/0	0/0	0/0	16.3/0	0/0	0/0	0/0
E13	0/0	0/0	0/0	0/20.	0/0	0/25.0	0/0	0/0	0/0
E25	0/0	0/20.0	11.1/0	0/0	0/12.5	8.3/0	0/0	0/0	9.0/0
E29	0/0	0/0	0/0	9.0/0	0/0	0/0	9.0/0	0/0	0/0
E30	0/0	0/20.0	11.1/0	9.0/20.0	7.1/0	0/25.0	0/0	0/0	0/0
E33	0/0	0/0	0/0	0/0	0/12.5	8.3/0	0/0	12.5/0	0/0
CB	0/16.6	30.0/20.0	22.2/0	9.0/0	14.3/12.5	25.0/0	9.0/25.0	25.0/0	18.2/25.0
PV	16.6/0	20.0/20.0	22.2/0	18.18/20.0	21.4/25.0	16.6/25.0	27.3/0	12.5/0	36.4/25.0
UT	25.5/33.3	10/0	11.1/40.0	36.3/20	35.7/12.5	8.3/25.0	45.5/0	50.0/75.0	36.4/50.0

*N/N* Total no. of Enteroviruses isolated from clinical/ sewage samples, *E* Echovirus, *PV* Poliovirus, *CBV* Coxsackie B virus, *UT* Untyped virus

### Yearly analysis (2007)

The quarterly report of sewage and clinical isolates during the month from July to September showed that the predominant enterovirus were E6, E9, and UT. E9 and UT was 33.3% in sewage whereas in clinically the percentage was 8.3 and 25.5 respectively. Human echovirus (16.6%) was detected clinically but absent in sewage. During the month from October to December, the three most common clinical and sewage EV were- E11, CB and PV however, E25 and E30 were detected in sewage but absent in clinical cases.

### Year 2008

In the first quarter (January to March), the CB- 22.2% and PV- 22.2%, were most commonly detected clinically but were not detected in sewage. UT was detected both clinically (11.1% of cases) and in sewage (40% of isolates). The E3 and E7 were also most commonly detected in sewage (25.0% for both isolates) but not in clinical cases. In the second quarter (April to June), the most common clinical EV, were UT, E9 and E30 (36.3%, 9.0% and 9.0 respectively), and their percentage was steady in sewage samples (20.0% for all of the isolates). E4, E29 and CB, observed frequently in clinical cases (9.0%, for all three isolates), but not detected in sewage. Human echovirus 13 was detected in sewage, but not in clinical cases. In third quarter (July to September), UT isolates were predominant in clinical cases of EV (35.7%) and also most commonly detected in sewage samples of EV (12.5%). Human echovirus 11 and CB, both were detected in clinical and sewage samples, whereas E25 and E33 were detected in sewage but not found in clinical cases. At the end of 2008 (October to December), UT was the predominant EV in both sewage and clinical samples, with 25.0% and 8.3% of the isolates respectively. Poliovirus accounted for 16.6% of the clinical cases and 25.0% of the sewage isolates and conversely, E9, E11, E12, E25, and E33 were detected in clinically but not in sewage isolates.

### Year 2009

The first quarterly information from January to March showed that CBs were more frequently detected in clinical and sewage samples (9.0 and 25.0% respectively). The echovirus 7, E29, and PV were detected clinically but not in sewage isolates. Percentage of all three isolates E4, E9, and E11 was similar to sewage, whereas these EV were not isolated in clinical samples. In the second quarter from April to June, the UT samples were predominant with high percentage (75.0) in sewage, while clinically it was 50%. The echovirus 33, CB, and PV were isolated clinically but not in sewage. However, E6 (25.0%) was detected in sewage, but was not found in clinical cases. In the third quarter from July to September, the UT was predominant in clinical and sewage isolate in 2009, with 36.43% of the clinical isolates and 50.0% of the sewage isolates. The echovirus 25 (9.0%) was found in clinical isolates, but was not found in sewage whereas both CB and PV were detected in clinical and sewage samples.

## Discussion

Drinking water is most important for transmission of EV from the epidemiological point of view [25]. These viruses are not easily inactivated and may persist in sewage for longer periods [26]. They are resilient organisms, able to withstand a high concentration of sodium chloride (NaCl) and large changes in temperature. These abilities allow the viruses to survive in water environment, their natural reservoir. In our study, Coxsackie viruses, untypable isolates, and serotypes of E9 and E11 were most frequently detected viruses throughout in clinical and sewage samples. E25 were present in sewage samples in the month from October to December 2007 which appears in clinical samples from January to March 2008, and again reappeared in sewage samples from July to August 2008, its circulation was observed in clinical samples in the last quarter of 2008. Similar kind of transmission was observed in E33, these were present in sewage samples from July to August 2008 and appeared in clinical samples in the next quarter of the year. This may be due to the fact that these viruses are ubiquitous in the environment. E12 serotype was present only in clinical samples in first and last quarter of the year 2008 whereas E13 was present in sewage samples only in the second and the last quarter of 2008. Enterovirus is stable for 1–3 h at pH of 3–5. They are neither susceptible to proteolytic enzyme nor to bile salts. Stability of the virus in external environmental conditions depends on temperature, humidity, and UV radiations. Exposure to daylight for 24 h has led to inactivation of 99.9% of PV [1, 27, 28]. A study conducted by Lodder et al. demonstrated that the local sewage which is being discarded into the river mass in Holland had very high density of enterovirus particles [23]. The study conducted in Holland revealed 14 positive results out of 64 [29], not only mussels, but any food that comes in contact with contaminated water and is prepared unhygienically can be a source of an epidemic outbreak. Third world countries and areas undergoing recurrent flooding are at greatest risk of developing enterovirus epidemics. Poor sanitary conditions and overcrowding are also causative factors. Studies conducted in South African countries and areas of Indonesia demonstrated that over 90% of children’s aged 5 have systemic antibodies proving contact with at least one enterovirus [30]. The inherent features of a tropical environment are a relatively constant, warm, and humid climate and the flora and fauna is determined by such a climate. These features probably exert little direct influence on the spread of enterovirus, since these sagents are very stable in the free State [31]. Clinical samples were drawn in case of acute infection, which resulted in increased detection of serotypes. Specimens from the environment like sewage is less concentrated and thus resulted decreased detection. Moreover, environmental samples are more exposed to extreme physiochemical conditions like pH, increased temperature, and exposure to UV light, desiccation etc. [32] that may result in inactivation of viruses, and thus decreased detection. This approach yields a comparatively more conservative prediction of the behavior of viruses in the environment.

A review by Gelfand makes it clear that these viruses as a class of ubiquitous and during the appropriate season, can be detected frequently in the feces of children, sewage, deprived sanitized areas, privy specimen and filth flies. The ability of virus spread is depends on the mode and duration of their excretion. Existing data for non-polio enterovirus indicates that they are excreted in the feces and probably in the pharynx [33]. Poor sanitation, great concentrations of humans, warm and humid climate, creates a new threat [24, 34]. Prevalence of virus in sewage water reflects the viral infections within the community, whether they are caused by wild viruses (coxsackieviruses, Human echovirus) or by vaccine viruses (poliovirus) [35]. Human enterovirus infection is generally known as asymptomatic, and thus, environmental surveillance has been reported to be a sensitive method to detect silently circulating viruses [6, 9]. Shorter-term comparative clinical and sewage studies in U.K. [6], Canada [8] and USA [9] had also demonstrated similar occurrence of Human echoviruses and type B coxsackievirus during the summer months. During our study period of 2 years, mainly untypable EV were found to be more frequent and predominantly present in environment as well as in clinical samples. Annual peaks of both sewage and clinical cases of EV occurred in late summer or early fall. In some years, early spring sewage EV signifies some of the EV that would predominate clinically during the following summer. EV that are detected in sewage can at times be predictors of some of the EV types that will be predominant clinically in the next coming season [9]. RT-PCR was carried out for untypeable samples by micro-neutralization test. Some previous reports suggested that RT-PCR aimed at 5’ UTR region is highly sensitive in EV detection from AFP patients, and suggested interspecies recombination concerning this region [36, 37]. The high epidemiological potential of enteroviruses calls for a surveillance of their circulation in the children’s communities, preferably by the virological monitoring of sewage. In addition to being effectively used to detect wild poliovirus transmission, environmental surveillance may become a powerful tool in the early detection of circulating vaccine-derived poliovirus strains [38]. Similar studies have been demonstrated in Netherlands [22] and Finland [39], for wild poliovirus surveillance. Such study can also be useful for monitoring of human echoviruses coxsackie viruses and other viruses which spread through contaminated water.

## Conclusion

In our study, we found that coxsackieviruses, untypable isolates, and serotypes of E9 and E11 were most frequently detected viruses throughout in clinical and sewage samples. There was a close relation among the stereotypes of enteroviruses shed in stools and isolated from the environment, but few serotypes which were detected in sewage samples were not found clinically and the few which were detected clinically not found in sewage because some viruses are difficult to detect by the cell culture method. To improve the correlation between sewage and clinical cases we can use the real time PCR method and also there is a need to increase surveillance system. The restricted enterovirus season began from July and peak season generally arises in September. This study will be helpful for the researchers as well as medical practitioners who are working on polio and nonpolio enterovirus especially in the countries which are struggling for polio eradication.

### Highlights

- Coxsackieviruses, untypable isolates, and serotypes of E9 and E11 were most frequently detected viruses throughout in clinical and sewage samples.
- There was a close relation among the stereotypes of enteroviruses shed in stools and isolated from the environment, but few serotypes which were detected in sewage samples were not found clinically and the few which were detected clinically not found in sewage because some viruses are difficult to detect by the cell culture method.
- To improve the correlation between sewage and clinical cases we can use the real time PCR method and also there is a need to increase surveillance system.

## Abbreviations

AFP: Acute Flacid Paralysis
CB: Coxsackievirus
CCID_50_: Cell culture infective doses
CPE: Cytopathic effect
DTT: dithiothreitol
E: Echovirus
EV: Enterovirus
FBS: Fetal bovine serum
MEM: Minimum essential media
NPEV: Non-polio enteroviruses
PEG: Polyethylene glycol
PV: Polio virus
RD: Rhabdomyosarcoma
TE: Tris EDTA
